# What is needed for continuity of care and how can we achieve it? – Perceptions among multiprofessionals on the chronic care trajectory

**DOI:** 10.1186/s12913-022-08023-0

**Published:** 2022-05-23

**Authors:** Linda Ljungholm, Anette Edin-Liljegren, Mirjam Ekstedt, Charlotte Klinga

**Affiliations:** 1 Department of Health and Caring Sciences, Linnaeus University, Pedalstråket 13, S-39182 Kalmar, Sweden; 2 Department of Epidemiology and Global Health, Umeå University, Umeå, Sweden; 3 The Centre for Rural Medicine, Research and Development Unit, Region Västerbotten, Storuman, Sweden; 4 Department of Learning, Informatics, Management and Ethics (LIME), Karolinska Institutet, Stockholm, Sweden; 5 Research and Development Unit for Elderly Persons (FOU Nu) Region Stockholm, Stockholm, Sweden

**Keywords:** Continuity of care, Integrated care, Healthcare organization, Conventional content analysis

## Abstract

**Background:**

Continuity of care (CoC) implies delivery of services in a coherent, logical and timely fashion. Continuity is conceptualized as multidimensional, encompassing three specific domains – relational, management and informational continuity – with emphasis placed on their interrelations, i.e., how they affect and are affected by each other. This study sought to investigate professionals’ perceptions of the prerequisites of CoC within and between organizations and how CoC can be realized for people with complex care needs.

**Methods:**

This study had a qualitative design using individual, paired and focus group interviews with a purposeful sample of professionals involved in the chain of care for patients with chronic conditions across healthcare and social care services from three different geographical areas in Sweden, covering both urban and rural areas. Transcripts from interviews with 34 informants were analysed using conventional content analysis.

**Results:**

CoC was found to be dependent on professional and cross-disciplinary cooperation at the micro, meso and macro system levels. Continuity is dependent on long-term and person-centred relationships (micro level), dynamic stability in organizational structures (meso level) and joint responsibility for cohesive care and enabling of uniform solutions for knowledge and information exchange (macro level).

**Conclusions:**

Achieving CoC that creates coherent and long-term person-centred care requires knowledge- and information-sharing that transcends disciplinary and organizational boundaries. Collaborative accountability is needed both horizontally and vertically across micro, meso and macro system levels, rather than a focus on personal responsibility and relationships at the micro level.

**Supplementary Information:**

The online version contains supplementary material available at 10.1186/s12913-022-08023-0.

## Background

A growing number of people worldwide live with complex care needs resulting from a combination of co-existing chronic health conditions, cognitive or functional impairment and/or social vulnerability [1]. This means that they require support from multiple healthcare and social care services to manage treatment, symptoms and activities in daily living [2]. A problem is that the current design of healthcare and social care systems does not meet the needs of people requiring multiple, integrated interventions. This applies to the population in general and older people in particular. In one study, between 29 and 51% of people in eleven OECD countries experienced problems with coordination between primary care and specialized care [3]. Cooperation between caregivers to achieve continuity of care (CoC) is deficient [4–6], despite CoC being one of the core qualities of primary care [7]. The descriptions, dimensions and concepts of CoC vary in the literature, with several converging and overlapping terms used, such as integration of care, patient-centred care, case management and coordination of care [7–12]. Previous research has shown that high performance regarding these concepts has a positive effect on patient experiences, care quality, coordination and the occurrence of cohesive care [13]. It has also become clear that care integration involves several challenges concerning organizational and managerial aspects, as well as aspects related to coordination and team collaboration [14, 15]. Another challenge is taking the needs of both patients and the employees into account [15] and dealing with organizational differences in how people work [16]. In the home care setting, it is suggested that the two dimensions – management of care and its delivery – need to be considered together as they interact and create a process of care experienced as continuous [17].

The Swedish healthcare system is decentralized, with legislation for healthcare policy being decided at the national level, while the 21 regions and 290 municipalities are self-governing and independent entities that provide healthcare. Healthcare in Sweden is generally funded by state and regional taxes and the regions are politically managed, with elections every four years [18].

The foundation for healthcare in Sweden, as for many other Western countries, is primary healthcare services (encompassing more than 1,100 care centres), providing medical treatment, rehabilitation, nursing, and preventive work for patients who do not require the technical and medical resources of a hospital. The responsibility of the regions and municipalities for provision of home healthcare services is regulated in the Health and Medical Services Act [19]. The municipalities are also responsible for meeting the homecare and housing needs of older people and people with disabilities, at all stages of life, in their private homes. These tasks are regulated in the Social Services Act [18, 20].

Since healthcare and social services are complex, governed by multiple organisations and laws, the ways in which CoC is delivered are many [21]. This also applies to how continuity is described. There is no consensus on the concept of CoC, but it is often described as being divided into three domains: relational, informational and management continuity [9]. Relational continuity can be described as an ongoing therapeutic relationship between a patient and one or more caregiver [9] or having a core team of professionals who know a patient and collaborate in patient care [7]. Informational continuity is described as the use of information to provide insight into previous care events to make current care suitable for everyone [9]. Informational continuity includes assessment of a patient’s needs, problems and resources and the patient’s values and the context [22]. Management continuity is described as a consistent and coherent approach to managing health conditions that is responsive to a patient's changing needs [9], as also encompassing consistency in clinical management [9, 23]. Management continuity includes standardizing care, when possible, through the implementation of guidelines and protocols [24]. However, clinical guidelines are based on evidence for optimal treatment of one disease at a time, which complicates the provision of joint decision-based patient-centred care, especially in caring of people with multimorbidity in primary care [25]. For these conditions, management continuity is achieved through effective multidisciplinary team collaboration across care boundaries in the provision and sharing of care planning and all necessary coordination of care that meets a patient's changing needs over time [26].

To counteract fragmentation of care, a conceptual framework was developed by Valentijn et al. [12], combining the functions of primary care with the dimensions of integrated care. According to this framework, both vertical and horizontal integration are needed. The dimensions of integrated care are structured at macro (system), meso (organizational) and micro (clinical) levels. To fit the needs of people across the continuum of care, the macro level must accommodate tailored combinations of structures, processes and techniques. At the meso level, services should be produced and delivered in a linked fashion and through collective action from the organizations throughout the care chain as they have joint responsibility for health and well-being. Professional integration is based on partnership between professionals, both within and between organizations. At the micro level, services should be adapted for coordination of person-focused care of patients across time, place and care discipline [12]. Many models and examples of care integration have been developed and modified to improve CoC, but little is known about what actually works [27–29]. One group that is particularly vulnerable is older people with multiple illnesses. Integrated care for older people focuses mainly on micro level clinical care integration processes, while information regarding meso and macro level care integration strategies is insufficient [30].

Delivery of integrated care to a growing population living with chronic conditions and complex care needs is challenging. Despite grand initiatives to bridge the gaps in CoC at the sharp end of practice for specific patient groups, this is far from realized. To achieve CoC for people with complex needs, we need to understand *what* prerequisites are needed for intra- and inter-disciplinary collaboration, both horizontally and vertically in the organizations, and *how* delivery of CoC should be managed. The purpose of this study was to investigate professionals’ perceptions of the prerequisites of CoC within and between organizations and how CoC can be realized for people with complex care needs.

## Methods

### Design

In order to capture a diversity of perspectives based on contextual variation, a qualitative cross-sectional study design was applied, using data from three different geographical areas in Sweden. Interviews were carried out with professionals within healthcare and social care services in a rural area in the north of Sweden, in the capital area, Stockholm, and in a smaller town and its surroundings in the southeast of Sweden.

### Study setting

Sweden has over 10 million inhabitants, living in 21 regions. The northern area, Norrbotten and Västerbotten, occupies approximately 40% of Sweden’s land area with a population of about 0.5 million inhabitants, 5% of the total Swedish population. Most of the inhabitants in the north live on the east coast, while the inland is very sparsely populated, approximately 1 inhabitant/km^2^. The informants in this study were from the inland. The urban area, Stockholm is Sweden’s most populous region, with just over 20 percent of the population, about 2.3 million inhabitants, and a population density of 328 inhabitants/km^2^. Kalmar region is situated in the southeast of Sweden and has the smallest population in the study: just over 245,000 inhabitants and a population density of 22 inhabitants/km^2^. The region encompasses Sweden’s second largest island, Öland, which is relatively sparsely populated.

In the three regions, healthcare provision faces differing circumstances and challenges. In Norrbotten and Västerbotten, one major challenge is the distances between inhabitants and healthcare centres and social care services. Inhabitants may have to travel up to 300 km to the nearest hospital with specialist functions and there is sometimes a lack of competence. It has been found that informants working in sparsely populated areas face other challenges regarding CoC than those working in large cities [31]. In Stockholm, the challenges are of a different kind – with many specialist hospitals and healthcare services, both public and private providers, to choose from. Kalmar is one of the regions in Sweden with the oldest population, with over 35% of the population being above 65 years old in some municipalities [32].

### Participants

This study is part of a larger project on CoC where patients with chronic conditions and their family carers were interviewed about their experiences in a first study [10]. In the current study, the initial sampling strategy was to recruit professionals along the chain of care for interviews by asking the patients in the first study to indicate their care contacts. It proved difficult to get in touch with the listed contacts, as most patients did not remember the professionals’ names. Seven out of 29 contacts were reached and willing to be interviewed. Therefore, we continued with a purposive sampling strategy [33] and recruited professionals across healthcare and social care services in the three regions. To gain as much insight as possible into how continuity is achieved for people with complex care needs from a cross-disciplinary perspective we strived to include a wide variety of care providers and professionals with as different roles and experiences of the aim under study as possible. The distribution of participants across different care providers and their roles in the three regions are presented in Table 1.

**Table 1:** Demographic data of study participants

Geographic area	RV/RN (*n*)	RS (*n*)	RK (*n*)
**Gender**			
Male	1	-	1
Female	8	9	15
**Interview method**			
Individual	9	9	3
Focus group	-	-	2
Paired	-	-	2
**Care provider**			
Municipal care^a^	3	1	14
Primary healthcare^b^	6	6	2
Specialist care^c^	-	2	1
Professions represented (n)	Registered nurse (16), Assistant nurse (9) Physiotherapist (4), Physician (3), Occupational therapist (1), Social worker (2)		
Specific roles represented (n)	Unit manager (4), Operations manager (1), Head of department (2), Patient safety and quality coordinator (1), Care coordinator (1)		

*RV/RN* Region Västerbotten/Norrbotten, *RS* Region Stockholm, *RK* Region Kalmar

^a^home care, home healthcare, social care services. ^b^ public primary care providers. ^c^ advanced home healthcare department and hospital

Different health and social care providers were contacted by the researchers located in each region by phone or in a personal meeting. The managers distributed verbal and written information about the study to professionals within different disciplines and roles and who – in accordance with the inclusion criteria – had broad experience and knowledge about coordination and performing care of people with complex needs. Professionals willing to participate were then contacted by one researcher in each region for an interview. All participants gave informed written consent.

In total, 34 informants were interviewed: one declined participation. The number of years in the profession ranged from 1 to 34 years (mean 16.4 years) and most of the informants were women in the healthcare sector with a nursing background. Quite a few had leadership positions and responsibility for specific areas at their workplaces (Table 1).

### Data collection

Multiple interview methods were used for data collection, adapted based on participant convenience, as a combination of methods can provide a more nuanced understanding [34]. In total, 25 interviews were conducted between October 2018 and November 2019. Twenty-one individual interviews and two paired interviews, defined as one researcher interviewing two people together, were held. Two focus group interviews were conducted, one with six healthcare professionals in connection with a workplace meeting and one with three participants from different levels in the chain of care. Two researchers (LL and ID) moderated the focus group discussions. The interviews were carried out by the authors (CK, AEL and LL) by phone (*n* = 14) or face-to-face (*n* = 11) at the participants’ workplaces (Table 1). All authors jointly developed a semi-structured interview guide with open-ended questions capturing the participants’ experiences and thoughts about what is needed to achieve CoC. The interview guide was built around thematic areas on organizational conditions, information transfer, and communication, the importance of the relationships with patients and team, and continuity as a concept (see Additional file 1: Appendix 1). The durations of the interviews were 17–60 min (median 32,43 min). All interviews were digitally recorded and transcribed verbatim.

### Data analysis

The interviews were analysed using conventional content analysis, as described by Hsieh and Shannon [35], enabling both data collection and analysis to be performed in iterative cycles. Another advantage of this method is the possibility of inductive category development, where researchers allow new insights and new categories to evolve from the data [35]. The software Microsoft® Excel was used for the analyses.

In the first step, all authors read the transcripts several times to get a sense of the whole. Then, a more thorough reading was carried out by CK, AEL and LL in order to highlight words and pieces of text that seemed to capture core components or concepts of CoC. This was interspersed with discussions of initial codes and notes and thoughts within all authors. The coding process continued with CK, AEL and LL developing a coding scheme. Codes that were related and linked, forming meaningful clusters, were organized into subcategories. During this phase, continuous dialogue took place between CK and AEL, resulting in a preliminary organization of the subcategories into a smaller number of categories. The subcategories were discussed with all authors and sorted into different levels (micro, meso and macro). Through iterative steps and a process of negotiated consensus [36], the categories and subcategories were developed and agreed upon jointly by all authors. All data were analysed together, although it was possible to track the region they originated from. Quotations from the transcripts were selected to illustrate the results. To enhance understanding, the translations into English given in this paper may deviate slightly from the verbatim transcripts.

## Results

The main results of this study were that the achievement of CoC was dependent on professional and cross-disciplinary relationships at the micro, meso and macro system levels, i.e., on intra- and inter-organizational cooperation. Relational continuity was the focus at the micro level (intra-organizational cooperation), whereas management continuity was found to be central at the meso level (intra- and inter-organizational cooperation) and the authorization of informational continuity was the focus at the macro level (inter-organizational cooperation). The three types of continuity were not mutually exclusive but were emphasized to varying degrees at the different levels. An illustration of the overall results is presented in Table 2 and an in-depth description of findings at each level is given in the text below.

**Table 2:** Findings of what is needed for CoC for people with complex care needs and how to achieve it at different levels of care

Level	What	How	Description
**Micro**^**a**^ **Intra-organizational** **Cooperation**	Long-lasting, customized relationships in team-based care	Set aside time to develop - needs-based - holistic relationships	Person-centred care, based on a holistic approach, where unique needs and (personal and environmental) resources determine how care will be designed and planned for the individual
		Provide - predictable and - accessible care with continuous follow-ups	
**Focus on relational continuity**			
**Meso**^**b**^ **Intra- and inter-organizational** **Cooperation**	Dynamic stability in organizational structures and routines for cross-disciplinary teams responsible for geographically defined areas	Establish teams - facilitating long-term patient care - coordination and responsibility	Adaptive and long-term ability of the organizations to handle change and continue to develop in accordance with ever-changing needs (the impression of stability depends on continuous adaptation to changing needs)
**Focus on management continuity**			
		Strive for - low staff turnover - clear professional roles - joint development of routines	
**Macro**^**c**^ **Inter-organizational** **Cooperation**	Long-term solutions that enable knowledge and information exchange and affirm shared responsibility for cohesive care	Support building of lasting inter-organizational cooperation based on - knowledge - trust - respect	Resource allocation enabling joint responsibility for cohesive care and establishment of shared information and communication platforms
**Focus on authorization of informational continuity**			
		Enable - regular cross-organizational information transfer - knowledge exchange	

^a^The micro level encompasses personal relationships, i.e., relationships between patients and professionals as well as between professionals at the clinical level

^b^The meso level encompasses management of healthcare services, i.e., organization of healthcare services and execution of work by professionals within and across organizations

^c^The macro level encompasses governance of the conditions for healthcare at the regional level, for example in terms of regulations and technical solutions for information and communication systems

### The micro level – intra-organizational cooperation

The micro level encompasses personal relationships, i.e., relationships between patients and professionals as well as between professionals within an organization.

#### What: Long-lasting, customized relationships in team-based care

The main aspects emphasized as essential to CoC for the patients, at the micro level, concerned relationships between professionals and patients, but also between professionals in cross-disciplinary teams. It was seen as important to have long-lasting and customized relationships between professionals in an organization and to have a holistic approach toward patients to achieve continuity.

#### How: Set aside time to develop needs-based and holistic relationships

According to the informants, relational continuity was enabled by providing patients the possibility to retain the same team of health professionals and by setting aside time to develop meaningful and needs-based relationships. Further, it was said that this gave the health professionals a better ability to get an overview, not only of the medical and health-related issues, but also of a patient’s entire life situation. Relational continuity includes mutual knowledge and understanding of each other, implying that the professionals should be aware of their patients’ history and diseases, current needs and individual and contextual resources. Mutual knowledge and understanding were also emphasized as providing conditions for patients to build trust and confidence in the professionals’ skills and assessments. Even a short encounter, such as in acute care situations, may build a relationship, and bring what matters to the patients to the surface. This information may be transmitted to the next instance and create continuity although a long-term relationship is not established. One of the informants described this as follows:

*That you see a person with a broken arm, not just a broken arm going home from the emergency room, for instance. […] that you have seen them and asked: ‘Who are you? What do you need? What might become difficult for you because this has happened to you?’* SLL_3

In general, the need to set aside time for the patients was highlighted as central, especially when a new relationship was being established. The informants emphasized the importance of having enough time to help the patients identify and express their needs and explore their abilities of self-care and participating in care planning. Time to prepare for a visit by reading the patient’s medical record was also emphasized as important for achieving continuity, along with personal skills like the ability to listen, affirm the patient and build trust during a visit. Moreover, a professional’s abilities to adapt communication to a patient’s needs and capabilities and be responsive to the patient’s wishes and expectations were highlighted as vital and linked to the amount of time available for a visit. The informants stated that giving patients the space to talk gave them a positive feeling as it facilitated the achievement of jointly set goals. Several informants expressed the need for time to allowing meaningful and needs-based relationships to grow:

*No, but that you feel that you have time to build some kind of relationship, so I can also understand what my patient expects me to do. Like, what they want out of it and what’s important to them, for instance, so you can do it as well as possible.* KLL FG

The informants highlighted several advantages from relationships growing over time. Such relationships meant that they could provide better care in emergency situations and treatments could start more promptly, as they had all the basic knowledge about these patients, making thorough investigations straightforward. Another advantage was that established relationships could promote psychological readiness for both professionals and patients. When a professional had knowledge of a patient, it was considered easier to notice needs that were not obvious.

*It’s easier for me to meet a patient […] that I know. It’s faster and more friction-free, and I can more easily realize things like, well, this doesn’t seem right with this patient, something must have happened.* VLL_7

From a patient safety perspective, relational continuity was emphasized as valuable, for instance in cases when a patient could not remember things or articulate their medical situation. Non-verbal communication was described as important. One of the informants described this as follows:

*It’s not just what is said […] there’s a lot of body language […] learning to understand what isn’t being said […] You learn to interpret what they mean and if the patient maybe has a family, you get to know the family and get a feel for the relationships within the family […] We need to map what resources we have in the patient’s surroundings.* SLL_5

The professionals’ understanding of the benefits expressed by their patients when given time to establish relational continuity was that they appreciated not having to recount their medical and life history at each new care encounter, i.e., that they did not have to repeat themselves. This led to patients feeling more relaxed and less insecure. Relational continuity promoted a better experience of care and increased the chance that patients felt confident in the professionals’ assessments. Another benefit of relational continuity was that it contributed to reducing the risk of a patient being given differing suggestions from different physicians with differing views of the patient’s health status. One informant highlighted a need for a warning system if a patient had more than a certain number of different care providers.

*When they have a lot of different care providers, then it’s much more difficult to get continuity. […] when you have […] a certain number of care providers, you should have someone who acts as a coordinator for you. […] If there was a warning system, like that when there are this many people involved […], who should take on the main responsibility now?* SLL_3

#### HOW: Provide predictable and accessible care with continuous follow-ups

Individualized and written information to patients on where to call and whom to turn to with questions, in conjunction with high telephone availability, was emphasized as essential to increase accessibility and a sense of security. Providing better opening hours all year round and predictability on care follow-up were likewise important for CoC, as was information about when interventions in the care process were needed and how they would be followed up. It was stated that interventions should be planned, preferably with professionals within the team who know the patient, as a way to increase the possibility for continuity.

*When you’re booking an appointment for instance […] with a district nurse for redressing a wound, like if you have a wound on your leg, well, you’ve been to my colleague X here seven times before, so then I’ll book an appointment with her, because they … well, you know … it’s much easier when you know one another.* KLL_1

It was highlighted that when patients had long-term contact with professionals in a team, the professionals could get an overview of the patient’s care, including which other professionals (both within and outside their own unit) were involved and the roles of any relatives. Moreover, the professionals were able to keep track of how treatment was progressing and if there had been any changes. In addition, when the professionals knew a patient’s history and what worked for him/her and could plan ahead, this allowed them not only to address the patient’s current situation, but also to go beyond superficial conversations.

*If the patient is always having to repeat what has been said or what has been done, there’s a risk […] that you never get any deeper, that you always come back to the same issue. You never get a deeper conversation. You don’t get any closer to the goal, what is the issue for this patient? You can’t follow up, which means the patient doesn’t feel seen. […] Continuity is achieved when you have created an understanding of what the patient has experienced.* SLL_3

### The meso level – intra- and inter-organizational cooperation

The meso level encompasses management of healthcare services, for example organization of healthcare services and execution of work by professionals.

#### WHAT: Dynamic stability in organizational structures and routines for cross-disciplinary teams

At the meso level, several aspects related to management of healthcare services aiming to achieve coordinated long-term patient care were highlighted, along with provision of health services based on cross-disciplinary teams.

#### HOW: Establish teams facilitating long-term patient care, coordination and responsibility

The informants stated that some patients described the existence of a long-lasting professional relationship with one physician, a nurse or other healthcare professional as the most important aspect of care continuity. The informants also described that other patients emphasized the importance of being offered team-based care.

*Yeah, no, continuity […] for me personally, it doesn’t have to be the same person all the time, just that there’s a good team involved […] that you can communicate with and that they feel safe with […] that there’s someone there for the patient or that knows about their situation.* SLL_6

One aspect emphasized by the professionals when striving for long-term patient care was the importance of taking responsibility for the bigger picture of each patient’s situation. This could be done by connecting all the involved professionals across organizational borders and care levels to create a common understanding of a patient’s needs, wishes and resources from different professional perspectives. The aim was to offer the right help from the right professional at the right time. One way to achieve this was by having regular multidisciplinary team meetings where team members across organizational borders in chains of care meet and distribute the tasks among themselves. It was stated by the informants that a well-prepared and documented plan for each patient’s care and the fact that routine care followed predetermined guidelines created less need for personal continuity. One positive side effect of teamwork that was emphasized was that it was experienced as making healthcare services less vulnerable to the absence of specific professionals due to sick leave or holidays. Teamwork, i.e., cooperation between professionals, could be facilitated by having geographically defined areas for healthcare services. At the same time, it was stated that this presupposed a willingness to work in cross-disciplinary teams, across organizations.

*Well, I work to ensure that we have […] the same area that I drive around in, which means some patients recur, but also that I get to know a lot of the home care staff, for instance, which makes the work much faster and easier and much, much better. You feel more confidence for one another when you know one another.* SLL_7

The informants also stated that physical proximity could create the conditions for spontaneous collaboration and optimal use of competence through knowledge exchange. However, a few key prerequisites for good teamwork were emphasized, including the professionals’ own desire to work in teams and a high degree of trust between team members. In addition, shared responsibility and having the courage to ask for help were seen as central for good teamwork which in turn created the conditions for CoC.

*We have a collaboration forum where we can … well, talk about shared patients. […] We try to have a close collaboration between [district nurses in home care and an occupational therapist] to … create an understanding for our different professional roles. And that’s a collaboration that you have to work on continuously. I think that … if you don’t understand each other, then it’s hard to work together.* VLL_9

The safety aspect of care was repeatedly emphasized by the informants. By providing long-term care for a smaller number of patients, the professionals gained a greater ability to notice changes in health status and evaluate if something was urgent. This enabled early action when needed. The number of professionals in teams working with a single patient was also discussed. The informants highlighted that improved planning could ensure that there were not too many team members working with one patient. Moreover, both internal and external teamwork improved performance, making it smoother and faster. Offering patients, the opportunity to make spontaneous visits and ask questions was emphasized as important and linked to the professionals’ ambition to create needs-based care and a sense of safety for patients.

*Like the people with poor hearing […] for instance, who have trouble communicating by phone, they can come by and then they’ll see, like, there’s my nurse, and you can give them five minutes […] they get the help they need. Otherwise, they’ll just stay at home and get sores unnecessarily, that become infected and so on.* SLL_2

The informants highlighted the importance of the role of the coordinator in planning joint healthcare with a high degree of continuity. The coordinator would take responsibility for gathering and sharing information. It was emphasized that the role should be filled by a professional, but not necessarily a physician. It was important that the coordinator could be available by telephone, guide patients through the healthcare system and coordinate care for those unable to do this themselves.

#### HOW: Strive for low staff turnover, clear professional roles and joint development of routines

The well-being of professionals was emphasized as significant and one aspect of this, mentioned above, was having cross-disciplinary teams responsible for geographically defined areas. However, this was not enough. Good conditions for all professionals must be realized to reduce the risk of high stress and pressure. This could help professionals get involved and create deep relationships. In addition, it was mentioned that this could promote knowledge transfer, better workflow and organizational development.

*Staff turnover is a huge challenge and then that there are no clear routines regarding how we communicate. […] That’s stressful, really stressful […] and it ruins development in the operations as well. […] It decreases the speed and quality of care. […] I think that everyone benefits from it [that staff remains]. Primarily because you can develop operations together and identify good work methods that benefit the patients. And you can only do that if the staff remains.* SLL_2

A circumstance that was highlighted as aggravating was a lack of dialogue about patient care among some professional groups, including nurses and physicians. This was perceived as complicating work and could lead to delayed interventions. The informants underlined the importance of clear roles and responsibilities and shared working procedures and routines, to ensure that everyone was working towards the same goals. It was of great importance that structures for information transfer and documentation and routines were discussed and developed together, and observed by all professionals. Many informants stated that a lack of shared routines made work more difficult.

*There are no work methods that ensure people work in the same way. When a person quits, none of their work methods will be remembered.* SLL_2

The need of co-creation was emphasized in a broader perspective as well. Thus, healthcare services need to be developed, taking account of the various professionals’ competencies, in order to be appropriate for patients’ holistic needs. Furthermore, joint planning among professionals, with patient participation, was considered to provide more predictable services, increase patient safety and promote more communicable care plans.

*Here, we work with participatory care, so we’ll meet […] the district nurse and district physician. And we have a planning session on what we should do with such and such [the patient]. And then we’ll document that, that we’ve had a conference.* VLL_4

Disease-specific reconciliations (e.g., for diabetes and dementia care), regular meetings and preparations for discharge from hospital are examples of collaborations between professionals serving to create more cohesive care. In the context of home care, finding time for inter-organizational collaboration was considered challenging, particularly for those mostly working evenings and weekends. Functional workflows based on teams were proposed as one way to manage the vulnerability that appears when building continuity tied to a specific professional and when several care providers are involved, all of whom have their own routines. The informants described it as follows:

*Yeah, that there is a workflow. There should be a plan for workflow, routines for the workflow and sufficient time to allow for the workflow.* VLL_7

*You need a form of workflow that is focused on functionality. Not on any one person, so it shouldn’t be down to you feeling comfortable and safe with the same nurse for twenty years. It should be based on work processes that are the same and are applied by everyone.* SLL_2

### The macro level – inter-organizational cooperation

The macro level encompasses the governance of conditions for healthcare at the regional level, for example in terms of legal regulations and technical solutions for information and communication systems.

#### WHAT: Long-term solutions that enable knowledge and information exchange and affirm shared responsibility for cohesive care

One fundamental aspect that was underscored as vital for CoC, at the macro level, was enabling care to flow without interruption. This required close and regular cooperation across organizational boundaries, both locally and regionally.

#### HOW: Support building of lasting inter-organizational cooperation based on knowledge, trust and respect

It is emphasized that lasting inter-organizational cooperation could be achieved through systematic joint meetings taking place on a regular basis. The informants mentioned that this could lead to trust and that respect-based reconciliations across levels were prioritized by all professionals. The primary aim of such meetings was to provide everyone involved in the care of a specific patient with up-to-date information. However, the importance of not scheduling unnecessary meetings and respecting everyone’s time was also mentioned.

*Yeah, in general, when I think about my profession [nurse], it feels like we sit there waiting to say what we need to say and then there’s usually a lot of other talk that is nothing to do with us, so it feels like we are wasting our time there sometimes.* SLL_2

The informants underlined the importance of constantly considering and working with quality by taking joint responsibility across healthcare services. This was described as having joint responsibility for overall care. However, it was also emphasized that responsibility must be matched by available resources. Everyone has an individual responsibility to get their own idea of a patient’s health situation – but everyone also shares a responsibility to find the best possible solutions for that patient where continuity has been considered.

*We have a very close collaboration with the municipality and home care and so on. And then when you consider region X […], it varies a bit between different units, I have to say. But I feel like people often pass the buck along … ‘That’s not my responsibility, that’s primary care’ and so on.* VLL_3

In line with what was mentioned regarding the micro level, the informants stated that trust needed to exist between professionals. One way to achieve a trustful cooperation was by having basic knowledge of other care providers and their interventions, routines and work methods, i.e., to have insight into how the various care providers work. Another way to achieve trustful cooperation was by gathering all the professionals at all the levels of care to reflect upon various issues based on different perspectives and competencies. Meetings for joint care planning was mentioned by the informants as an important arena, where providers could get insights into each other’s work and learn how other care providers reasoned regarding specific patients’ health. Such meetings require that care providers have permission to exchange information. Moreover, the importance of not forgetting anyone was underlined – everyone working with a patient’s care should be involved in such meetings. Generally, the informants described lacking knowledge about each other’s areas of expertise as negative for continuity, though they stated that most professionals had a generous attitude as regards sharing their knowledge.

*And then I also think about the knowledge within the professions, what responsibilities does […] a nurse in primary care have? What responsibilities does a physical therapist in the municipality have? […] You don’t really know, which makes it very difficult to know […] who you should call on to get the right kind of help.* KLL_3

Keeping high-quality medical records across healthcare services was highlighted as central by the informants. Transparency in other caregivers’ interventions was also emphasized as important from a patient safety perspective, as it enabled digital knowledge transfer between professionals at different levels of care if a patient’s health situation were to change. Moreover, the informants stated that this enabled better coordination of care visits and thus also better continuity. However, it was considered difficult to get an overview, as medical records often contained a lot of information, as described by one informant.

*Everyone writes a note in the medical record. The physician, the nurse, the physical therapist, everyone, and they are added in a chronological order, so if the patient has been here for a week, there can easily be twenty to thirty notes […] so it’s hard to get an overview.* KLL_6

#### HOW: Enable regular cross-organizational information transfer and knowledge exchange

Good communication and inter-organizational cooperation were highlighted as a shared responsibility for all professionals involved in the care of a patient. To increase CoC, the need of information transfer through shared systems and communication channels was emphasized. This would increase the opportunities to take long-term responsibility for coherent care. Informants in the northern regions highlighted challenges with coordination of care related to the large geographical distances between different caregivers, who seldom had the same information and communication systems.

*The most difficult part is when we have to work across regional borders. When there are patients with certain diagnoses, particularly some cancers, they get treatment at the specialist hospital, and they are managed by the medical clinic here and by primary care here and … so there are actually four care providers involved, with one being in a different region. That makes it … it’s hard to get the coordination to work.* VLL_5

In general, improved collaboration and communication between professionals was emphasized as contributing to rapid coordination and increased understanding of each other’s roles. This could be achieved through direct communication by telephone or via digital tools. One example of when accurate and timely information in medical records was of great importance was when patients were being transferred from one level of care to another, e.g., between primary care and a hospital. In these situations, quick access to information of the situation was seen as necessary for provision of appropriate help. One informant described a situation where this had not been possible:

*I had a patient last week who I sent to the care centre. […] I called […] beforehand, it was fairly urgent. In my view, the patient couldn’t wait until the next scheduled appointment, because she had gotten worse. So she got a new appointment […] and of course I was left wondering […] how has it gone? Have there been any changes that I need to know about, for instance in her medication? And when I looked in the medical records, no notes had been added yet. […] Later, I found out that the patient had been given new medication, but that was only after a few days. In this case, the patient had gone to the pharmacy and knew that she was supposed to take the medication in the evening, but I am responsible for her care and am supposed to sort it [the medication] into a pill organizer.* KLL_2

The informants stated that short, efficient reconciliations could improve workflow by preventing unnecessary difficulties or delays. Health information technology was mentioned as a suitable communication method, as it could enable meetings between professionals regardless of physical distance. Still, the need to adapt digital meetings to individual persons’ abilities and skills was highlighted. This applied both between professionals and between professionals and patients. Generally, the informants expressed a desire to achieve inter-organizational information transfer and knowledge exchange.

*… there should be a shared documentation platform, because then you could see current care contacts and what they are related to, regardless of where you are requesting care. Because there are different kinds of problems, and that would make it easier, because the professional who is looking at a record can see, like, what is going on right now and what contacts does this patient already have? … that would be good […] if it could encompass both municipal healthcare, home care and social care services … that would make it easier to get an overview.* KLL_3

## Discussion

This study investigated professionals’ perceptions of the prerequisites of CoC for patients within and between organizations and how CoC for people with complex care needs can be realized. Three levels where continuity is needed were identified: the clinical level, the professional and organizational level and the system level. This study contributes to broadening the understanding of CoC for patients by highlighting prerequisites for and execution of continuity at each level. At the micro level, which encompasses cooperation with patients and between professionals, the focus was on relational continuity. Management continuity was found to be central at the meso level, for achievement of intra- and inter-organizational cooperation. Informational continuity was the main focus at the macro level, which encompasses regional governance of inter-organizational cooperation. The three types of continuity were not mutually exclusive, but were highlighted to differing degrees in relation to the different levels.

The rainbow model, developed by Valetijn et al. [12], highlights the complementary roles of vertical and horizontal integration at the micro, meso and macro levels and the understanding of inter-relationships among the dimensions of integrated care. As the prerequisites and execution of CoC are vital for integrated care, we believe that the rainbow model is useful as a theoretical frame in discussing the findings.

### Micro level

At the micro level, clinical integration refers to how well services are connected and how coherent care provision to the individual patient can be. The findings suggested that this could be achieved by setting aside time to develop holistic and needs-based relationship with professionals working in intra-organizational teams. For patients, continuity is gained through trustful relationships and mutual understanding developed over time with a person or team who knows them well (relational continuity) [10, 37]. Research indicates that personal continuity (i.e., meeting the same physician or team of professionals) is associated with fewer complications and hospitalizations [38] and lower mortality [39]. Moreover, it increases shared decision-making [40–42], patient satisfaction and the provision of personalized and timely care [43]. Our study contributes with a complementary perspective indicating that person-focused holistic care, where the services match each patient’s unique needs and resources, is required. To fully understand the breadth of needs and resources, assessment of physical, mental, social, and environmental needs and personal preferences and goals is recommended [1].

Pursuing shared goals, through jointly agreed routines and guidelines for collaboration and evidence-based decision support, especially in work close to the patient, would promote continuity. Participants also highlighted that predictable and accessible care with continuous follow-up is essential for achieving continuity. Establishing shared goals has a coordinating effect [44], as it requires clearly defined responsibility and roles and active work from all the parties involved, including patients and family carers. In the management of chronic diseases, the focus has been on developing guidelines to implement standardized care for each disease. However, an approach based on single-disease recommendations may be inappropriate for people with multiple diseases and complex care needs, where a large number of care providers and professionals are involved in care. Then it becomes essential to establish an agreement on shared goals and a plan for achieving them, in line with the overarching goals set by the patient [45]. Professionals in this study suggests that although it is possible to address different diagnoses and challenges in separate plans, a unified plan may help to ensure predictability and feasibility in the patient’s life. This would require a system redesign that allows sharing of information between care providers. In highly fragmented healthcare systems, information tends to flow in separate pipelines between professionals at different system levels [4, 46]. This may result in competing and poorly coordinated subprocesses, which can be directly counterproductive for the patient. Other studies have also shown that shared information is essential for achieving a common ground for understanding between team members, enabling personalized care planning [47, 48]. It is also important to include monitoring and evaluation of the care process regarding these goals.

### Meso level

In line with the findings of Valentijn et al. [12], both organizational and professional aspects were found to be important when creating CoC at the meso level. Several characteristics of management continuity were critical for intra- and inter-organizational cooperation [9]. For instance, dynamic stability in organizational structures and routines was described as essential for cross-disciplinary teams and for achieving CoC. Such stability, based on guidelines and standards that enable the cross-disciplinary teams to perform equal care, also depends on the adaptive capacity of management and professionals in response to changing needs. For example, adaptations were described in the form of flexible scheduling, placing the right professional skills at the right place and at the right time. Further, establishment of teams was suggested to facilitate long-term patient care, coordination and accountability. Strong cross-disciplinary team collaboration was suggested to improve continuity and reduce stress to professionals [15]. However, teamwork is multifaced and complex, requiring that team members are willing to adapt to the needs in each situation [49]. For this reason, small teams in which one team member takes on a coordinating role were recommended in a study by Gjevjon and colleagues [15].

Management continuity has proven to be particularly challenging when it comes to coordinating actions with other care providers to deliver services in a complementary and timely manner along a patient’s care pathway [1, 9]. For healthcare professionals, continuity in this situation means having all the necessary information about the patient at the point of care. However, it also means having all the necessary information about the receiving organization’s competence, capability and routines, in order to provide appropriate information, preparations and equipment. Having designated care coordinators at both the clinical level and the management level would facilitate information-sharing. At the clinical level, a nurse or care coordinator assigned to guide the person with complex needs through the system would be warranted [19, 50]. In other settings, coordination at the management level has been shown to support coherent goal-setting among professionals, enabling them to achieve short-term goals, while not losing sight of the long-term overarching goals [51]. Our results reveal organizational preconditions that may facilitate management continuity, such as low staff turnover, clear professional roles and agreement on routines and standards. Sangaleti [52] has indicated that collaboration improves CoC, but requires communication, togetherness, humility and knowledge of different methods and professional roles. Still, high staff turnover may jeopardize CoC. Like Parker et al. [21], we believe that an appropriate culture, modern communication systems and dynamic management practices are needed to retain competent staff.

### Macro level

To create welfare systems that enable CoC for people with complex needs, traditional professional and organizational gaps need to be bridged. As suggested by the informants in this study, one way could be by creating long-term, uniform solutions that enable knowledge and information exchange and affirm shared responsibility for cohesive care. System-wide policies, laws and guidelines are needed to create conditions that facilitate coordination across organizational boundaries and administrative systems [25].

Valentijn suggests both vertical (disease-oriented) and horizontal (holistic, person-focused) integration of the highly complex healthcare developed to treat one condition at a time, in order to meet the complex and long-term bio-psychosocial and environmental needs of a multi-diseased person [12]. The dominating medical view on diseases needs to be complemented by a person-focused perspective on health that simultaneously acknowledges social and psychological needs and manages medical and social problems. The results of this study indicate that CoC can be achieved if several conditions coexist and cooperate at all levels, both vertically (across levels of specialization) and horizontally (across sectors) [12]. According to the informants, this could be achieved through systems that enable regular information transfer and knowledge exchange across both vertical and horizontal boundaries. However, a considerable challenge – also emphasized in other studies – is that healthcare and social care services occur in different sectors, governed by different laws and regulations, sometimes with conflicting goals, which can counteract cooperation [53]. Further, ethical concerns regarding personal integrity and laws on data sharing complicate inter-organizational information transfer. Lasting inter-organizational cooperation based on knowledge, trust and respect, has been suggested by our informants as essential for management and delivery of CoC across healthcare and social care services.

This study supports other research [54] emphasizing that CoC can be achieved through shared decision-making and alignment of goals. This could be realized through frequent joint meetings and reconciliations with professionals at all levels, coordinating visits, having basic knowledge of other care providers’ interventions, routines and work methods, and promoting accountability across organizational borders. Additionally, this study illustrates that even if one does not achieve personal continuity, CoC can arise through staff working in teams with common goals and routines, providing person-focused holistic care.

### Methodological considerations

Different methods were used for collection of data, with focus groups, paired and individual interviews all used to get richer data [34]. All data were analysed in the same way, with a focus on the aim. A combination of methods can lead to fewer rejections or withdrawals [34]. To increase credibility the recruitment of informants was considered to cover as wide variation as possible in the country. Participants chosen represented various professions, ages, genders and experiences who could describe their perceptions of what is needed for achieving CoC for people with complex care needs [55]. A drawback may be that there were biases in the distribution of participants among care providers, professions, and roles. On the chronic care trajectory, nurses are represented across all care contexts and in several roles, which contributed to high recruitment of nurses. Time pressure and staff shortages also constrained the recruitment of physicians and physiotherapists, for example. The quality and richness of the data obtained determines whether information power is reached [56]. In this study, we believe that the few interviews with physicians, occupational therapists, physiotherapists and social workers contributed to a more complete picture of cross-disciplinary collaboration and provided a complementary angle on what continuity may be and how it is achieved. Professionals at the macro level were not included in the sample and the results at the macro level are based on information from professionals at the micro and meso levels. Different steps were undertaken to establish credibility. The research group encompassed members with a range of professional backgrounds (registered nurse, social worker and biomedical scientist), providing differing perspectives on the data. Researchers self-reflected on own pre-understandings and discussed interpretations in relation to the informants' experiences with the research group. As three researchers were conducting interviews in the three settings, a semi-structured interview guide was developed, that was followed as thoroughly as possible to ensure stability in data collection. Conformability and trustworthiness was improved through systematic comparisons and repeated discussions about data and codes. Authencity was ensured through rich quotations from informants illuminating a range of reality [35, 55]. The results at the different levels in Table 2 covers the data that can be observed from the citations described in the result section. The context has been described in detail in the method section, to increase transferability to similar healthcare systems.

### Suggestions for future research and implications for practice

Prerequisites in terms of knowledge and understanding need to exist at the micro, meso and macro levels for continuity to be created for people with complex care needs. This study describes not only what the preconditions are, but also how CoC can be achieved, including some practical guidance. Further research is required to deepen knowledge of if and how best practices at the meso level can be achieved through development of methods and collaborative procedures.

## Conclusions

The achievement of CoC for patients with complex care needs was found to be dependent on professional and cross-disciplinary cooperation at the micro, meso, and macro levels.

At the micro level, the relational aspects of continuity were highlighted. They included enabling long-lasting and person-centred relationships between patients and professionals in cross-disciplinary teams using a holistic approach. In practice, professionals could set aside time to develop holistic and needs-based relationships and provide predictable and accessible care with continuous follow-up. At the meso level, the management aspects of continuity comprised dynamic stability in organizational structures and routines for cross-disciplinary teams responsible for geographically defined areas. In practice, the establishment of organizational conditions for cross-disciplinary teams facilitating long-term patient care and coordination responsibility was found to be important. This included efforts to decrease staff turnover, clarify professional roles and jointly develop routines. At the macro level, the informational aspects of continuity were found to relate to long-term, uniform solutions that enabled knowledge and information exchange and affirmed shared responsibility for cohesive care. Support to build inter-organizational cooperation based on knowledge, trust and respect and to enable recurrent cross-disciplinary information transfer and knowledge exchange was highlighted as important.

## Supplementary Information

**Additional file 1: Appendix 1.** Interview guide.

## Data Availability

The datasets used and/or analysed during the current study are available from the corresponding author on reasonable request.

## Abbreviation

CoC: Continuity of care

## References

[1] Kuluski K, Ho JW, Hans PK, Nelson MLA (2017). Community care for people with complex care needs: bridging the gap between health and social care. Int J Integr Care..

[2] Barnett K (2012). Epidemiology of multimorbidity and implications for health care, research, and medical education: a cross-sectional study. Lancet.

[3] OECD. Realising the Potential of Primary Health Care, OECD Health Policy Studies, OECD Publishing Paris [Internet] 2020. [cited 2022 jan 10]. Available from 10.1787/a92adee4-en

[4] Kneck A, Flink M, Frykholm O, Kirsebom M, Ekstedt M (2019). The information flow in a healthcare organisation with integrated units. Int J Integr Care.

[5] Flink M, Ekstedt M (2017). Planning for the discharge, not for patient self-management at home - an observational and interview study of hospital discharge. Int J Integr Care.

[6] Oksholm T, Rustoen T, Ekstedt M (2018). Transfer between hospitals is a risk situation for patients after lung cancer surgery. Cancer Nurs.

[7] Uijen AA, Schers HJ, Schellevis FG, van den Bosch WJHM (2012). How unique is continuity of care? A review of continuity and related concepts. Fam Pract.

[8] Gulliford M, Naithani S, Morgan M (2006). What is 'continuity of care'?. J Health Serv Res Policy.

[9] Haggerty JL, Reid RJ, Freeman GK, Starfield BH, Adair CE, McKendry R (2003). Continuity of care: a multidisciplinary review. BMJ.

[10] Ljungholm L, Klinga C, Edin-Liljegren A, Ekstedt M (2021). What matters in care continuity on the chronic care trajectory for patients and family carers? - A conceptual model. J Clin Nurs..

[11] Bahr SJ, Weiss ME. Clarifying model for continuity of care: A concept analysis. Int J Nurs Pract. 2019;25(2):e12704. 10.1111/ijn.12704. Epub 2018 Nov 4. PMID: 30393894. 10.1111/ijn.12704 30393894

[12] Valentijn PP, Schepman SM, Opheij W, Bruijnzeels MA (2013). Understanding integrated care: a comprehensive conceptual framework based on the integrative functions of primary care. Int J Integr Care.

[13] Adler R, Vasiliadis A, Bickell N, Adler R, Vasiliadis A, Bickell N (2010). The relationship between continuity and patient satisfaction: a systematic review. Fam Pract.

[14] Van Servellen G, Fongwa M, Mockus DE (2006). Continuity of care and quality care outcomes for people experiencing chronic conditions: a literature review. Nurs Health Sci.

[15] Gjevjon ER, Romøren TI, Kjøs BØ, Hellesø R (2013). Continuity of care in home health-care practice: two management paradoxes. J Nurs Manag.

[16] Hellesø R, Lorensen M (2005). Inter-organizational continuity of care and the electronic patient record: a concept development. Int J Nurs Stud.

[17] Woodward CA, Abelson J, Tedford S, Hutchison B (2004). What is important to continuity in home care? Perspectives of key stakeholders. Soc Sci Med.

[18] Anell A, Glenngård AH, Merkur S (2012). Sweden health system review. Health systems in transition..

[19] Health and Medical Services Act (2017:30) https://www.riksdagen.se/sv/dokument-lagar/dokument/svensk-forfattningssamling/halso--och-sjukvardslag-201730_sfs-2017-30.

[20] Social Services Act (1980:620) https://www.riksdagen.se/sv/dokument-lagar/dokument/svensk-forfattningssamling/socialtjanstlag-1980620_sfs-1980-620

[21] Parker G, Corden A, Heaton J (2011). Experiences of and influences on continuity of care for service users and carers: synthesis of evidence from a research programme. Health Soc Care Community.

[22] Freeman G, Hughes J. Continuity of care and the patient experience: An Inquiry into the Quality of General Practice in England. London: The king`s Fund; 2010. Available from: https://www.kingsfund.org.uk/sites/default/files/field/field_document/continuity-care-patient-experience-gp-inquiry-research-paper-mar11.pdf

[23] Reid R, Haggerty J, McKendry R. Defusing the confusion: concepts and measures of continuity of health care. 2002.

[24] Guthrie B, Saultz J, Freeman G, Haggerty J (2008). Continuity of care matters. BMJ.

[25] Wallace E, Salisbury C, Guthrie B, Lewis C, Fahey T, Smith SM (2015). Managing patients with multimorbidity in primary care. BMJ.

[26] Saultz JW (2003). Defining and measuring interpersonal continuity of care. Ann Fam Med..

[27] Berglund H, Wilhelmson K, Blomberg S, Dunér A, Kjellgren K, Hasson H (2013). Older people's views of quality of care: a randomised controlled study of continuum of care. J Clin Nurs.

[28] Berglund H, Blomberg S, Dunér A, Kjellgren K (2015). Organizing integrated care for older persons: strategies in Sweden during the past decade. J Health Organ Manag.

[29] Reed J, Cook G, Childs S, McCormack B (2005). A literature review to explore integrated care for older people. Intl J Integr Care.

[30] Briggs AM, Valentijn PP, Thiyagarajan JA, Araujo de Carvalho I (2018). Elements of integrated care approaches for older people: a review of reviews. BMJ Open..

[31] Ryuichi O, Yoshinori R, Jun K, Tatsunosuke G, Takuji K (2020). Challenges and solutions in the continuity of home care for rural older people: A thematic analysis. Home Health Care Serv Q.

[32] State public investigations (SOU2002:29) https://www.regeringen.se/49b6bb/contentassets/f985ca13c427488291d0f8d80d2d0264/del-2-t.o.m.-kap.-4

[33] Patton MQ (2002). Qualitative Research & Evaluation Methods: Integrating Theory and Practice. 3rd ed. Qualitative research and evaluation methods.

[34] Lambert SD, Loiselle CG (2008). Combining individual interviews and focus groups to enhance data richness. J Adv Nurs.

[35] Hsieh HF, Shannon SE (2005). Three approaches to qualitative content analysis. Qual Health Res.

[36] Bradley EH, Curry LA, Devers KJ (2007). Qualitative data analysis for health services research: developing taxonomy, themes, and theory. Health Serv Res.

[37] Waibel S, Henao D, Aller M-B, Vargas I, Vázquez M-L (2012). What do we know about patients’ perceptions of continuity of care? A meta-synthesis of qualitative studies. Int J Qual Healthcare.

[38] Hussey PS, Schneider EC, Rudin RS, Fox DS, Lai J, Pollack CE (2014). Continuity and the costs of care for chronic disease. JAMA Intern Med.

[39] Baker R, Freeman GK, Haggerty JL, Bankart MJ, Nockels KH (2020). Primary medical care continuity and patient mortality: a systematic review. Br J Gen Pract..

[40] Saultz JW, Albedaiwi W (2004). Interpersonal continuity of care and patient satisfaction: a critical review. Ann Fam Med.

[41] Katz DA, McCoy K, Sarrazin MV (2014). Does improved continuity of primary care affect clinician-patient communication in VA?. J Gen Intern Med.

[42] Rodriguez HP1, Rogers WH, Marshall RE, Safran DG (2007). The effects of primary care physician visit continuity on patients' experiences with care. J Gen Intern Med..

[43] den Herder-van der Eerden M, Hasselaar J, Payne S, Varey S, Schwabe S, Radbruch L (2017). How continuity of care is experienced within the context of integrated palliative care: a qualitative study with patients and family caregivers in five European countries. Palliat Med..

[44] Berntsen G, Strisland F, Malm-Nicolaisen K, Smaradottir B, Fensli R, Røhne M (2019). The evidence base for an ideal care pathway for frail multimorbid elderly: combined scoping and systematic intervention review. J Med Internet Res.

[45] Salisbury C, Man MS, Bower P, Guthrie B, Chaplin K, Gaunt DM, Brookes S, Fitzpatrick B, Gardner C, Hollinghurst S, Lee V, McLeod J, Mann C, Moffat KR, Mercer SW (2018). Management of multimorbidity using a patient-centred care model: a pragmatic cluster-randomised trial of the 3D approach. Lancet.

[46] Wibe T, Ekstedt M, Helleso R (2015). Information practices of health care professionals related to patient discharge from hospital. Inform Health Soc Care.

[47] Freilich J, Nilsson GH, Ekstedt M, Flink M. "Standing on common ground" - a qualitative study of self-management support for patients with multimorbidity in primary health care. BMC Fam Pract. 2020;21(1):233. 10.1186/s12875-020-01290-y. PMID: 33203401; PMCID: PMC7670978. 10.1186/s12875-020-01290-y PMC7670978 33203401

[48] Barnet M, Shaw T (2013). What do consumers see as important in the continuity of their care?. Support Care Cancer.

[49] Johansson G, Eklund K, Gosman-Hedström G (2010). Multidisciplinary team, working with elderly persons living in the community: a systematic literature review. Scand J Occup Ther.

[50] Patient Data Act (2008:355) https://www.riksdagen.se/sv/dokument-lagar/dokument/svensk-forfattningssamling/patientdatalag-2008355_sfs-2008-355.

[51] Hybinette K, Pukk Härenstam K, Ekstedt M (2021). A First-line management team’s strategies for sustaining resilience in a specialised intensive care unit—a qualitative observational study. BMJ Open.

[52] Sangaleti C, Schveitzer MC, Peduzzi M, Zoboli E, Soares CB (2017). Experiences and shared meaning of teamwork and interprofessional collaboration among health care professionals in primary health care settings: a systematic review. JBI Database System Rev Implement Rep.

[53] Goodwin N, Dixon A, Anderson G, Wodchis W (2014). Providing integrated care for older people with complex needs: lessons from seven international case studies.

[54] Berntsen GKR, Gammon D, Steinsbekk A, Salamonsen A, Foss N, Ruland C (2015). How do we deal with multiple goals for care within an individual patient trajectory? A document content analysis of health service research papers on goals for care. BMJ Open.

[55] Elo S, Kääriäinen M, Kanste O, Pölkki T, Utriainen K, Kyngäs H (2014). Qualitative Content Analysis: A Focus on Trustworthiness. SAGE Open.

[56] Malterud K, Siersma VD, Guassora AD (2016). Sample Size in Qualitative Interview Studies: Guided by Information Power. Qual Health Res.

[57] World Medical Association. Declaration of Helsinki [Internet]. 2013 [cited 2021 March 29]. Available from: https://www.wma.net/policies-post/wma-declaration-of-helsinki-ethical-principles-for-medical-research-involving-human-subjects/.

