# Supplementary material for: What is needed for continuity of care and how can we achieve it? – Perceptions among multiprofessionals on the chronic care trajectory
Source: BMC Health Serv Res. 2022 May 23;22:686. doi: 10.1186/s12913-022-08023-0 (PMC9125858; doi:10.1186/s12913-022-08023-0)
Supplement: Supplementary file 1 — Additional file 1: Appendix 1. Interview guide. [file 12913_2022_8023_MOESM1_ESM.docx]

# Appendix 1 - Interview guide

**Introduction to the interview:** The purpose of this study is to identify "best-practice" for continuity of care for people with complex care needs* in regions and municipalities. We want to interview you to get your perceptions **of** how care is coordinated for patients with complex care needs **who** you have met and **what continuity is** for you in your work.

*By complex care needs, we mean people with co-existing chronic health conditions, cognitive or functional impairment and/or social vulnerability, and/or that the person requires coordinated care from multiple healthcare and social care services to manage treatment, symptoms, and activities in daily living.

1. **Opening question:** How long have you worked as a/an …?
2. **Continuity of care as a concept**:

What does continuity mean to you? How would you define the meaning of continuity? What is needed for you to experience that there is continuity of care? Which aspects do you think are important? Do you work actively to create continuity in your work? If so, how?

1. **Organizational conditions:** Do you feel that the organizational circumstances for creating continuity are at hand? If so, what are they? If not, what would need to change in order to create better conditions for continuity? What do you think of the governing regulations in place today? Do they contribute to facilitating/impeding continuity?
2. **Information transfer and communication:** How do you feel that information transfer and communication work between you and patients and next-of-kin? What do you consider to be facilitating and impeding factors, respectively?

How do you feel that information transfer and communication work between other care providers? How does this affect your work? Do you feel that the existing systems for information transfer and communication with other parties are satisfactory? What would be the ideal situation, is there anything that could be improved/facilitate your work across organizational boundaries as regards the creation of continuity?

1. **Contact between care providers**: How much contact do you feel that you as care providers have with one another? What do you do to coordinate care? What coordination possibilities do you have for an individual patient?
2. **Care providers’ knowledge of the interventions of others and their own participation**: How much knowledge do you have about the interventions a patient receives from other care providers? (*if clarification is needed: what knowledge does the primary care center have about your rehabilitation exercises, for instance?*) How does it affect your work that you have (or do not have) knowledge of one another’s interventions? What do you do to ensure that other care providers know about your interventions?
3. **Valuation of continuity among staff**: How do you value the continuity in the contact with other care providers in your professional role? What importance does continuity in patient contacts have for you as a staff member? Does patient continuity impact on your work and, if so, how?
4. **The importance of the relationships:** What does a relationship with a patient mean to you? What is important to you in the relationship with the patient? How do you create a relationship? What do you do to create a sense of security in a relationship with a patient? What importance does continuity have for the relationship?
